# Is ‘better’ enough? Prevalence and multidimensional portrait of persistent dyspnea upon discharge from a respiratory medicine ward: a prospective, single-center observational study

**DOI:** 10.1186/s12890-025-03923-3

**Published:** 2025-10-06

**Authors:** Capucine Morélot-Panzini, Safaa Nemlaghi, Morgane Faure, Laure Serresse, Thomas Similowski

**Affiliations:** 1 UMRS1158 Neurophysiologie Respiratoire Expérimentale et Clinique, Sorbonne Université, INSERM, Paris, F-75005 France; 2 Service de Pneumologie (Département R3S), AP-HP, Groupe Hospitalier Universitaire APHP-Sorbonne Université, Hôpital Pitié- Salpêtrière, Paris, F-75013 France; 3 Fédération Hospitalo Universitaire “BREATH”, Paris, France; 4 Service de Soins Palliatifs, Soins d’Accompagnement et Soins de Support, AP-HP, Groupe Hospitalier Universitaire APHP-Sorbonne Université, Paris, F-75013 France; 5 Département R3S, AP-HP, Groupe Hospitalier Universitaire APHP-Sorbonne Université, Hôpital Pitié- Salpêtrière, 47-83 boulevard de l’Hôpital, Cedex 13, Paris, 75651 France

**Keywords:** Dyspnea, Hospital discharge, Chronic obstructive pulmonary disease, Asthma, Pneumonia, Lung cancer

## Abstract

**Background:**

During unplanned respiratory-related hospitalizations, gradual improvement in physiological variables and reduced dependence on treatment are crucial for discharge decisions, possibly supported by discharge care bundles designed to reduce post-hospitalization readmission and mortality. However, patients prioritize symptom relief. This study tested the hypothesis that a significant proportion of patients admitted to a pulmonology ward for an acute respiratory episode experience dyspnea on the day of discharge. It further aimed to describe this dyspnea in a multidimensional manner.

**Methods:**

This 10-week prospective study was conducted at a single center and included patients admitted for acute respiratory conditions such as COPD or asthma exacerbation, pneumonia, pulmonary embolism, or pleural disease, who, on admission, reported a rating of 3 or higher on the “immediate breathing discomfort” item of the Multidimensional Dyspnea Profile (MDP-A1). Dyspnea was assessed both at admission and at discharge using a multidimensional recall-based tool (MDP) and an instant unidimensional tool, the 10-cm visual analog scale (D-VAS).

**Results:**

Seventy consecutive patients were included in the study. Although dyspnea ratings showed a statistically significant decrease during the hospital stay, dyspnea remained both frequent and intense at discharge. At discharge, 84% of patients provided MDP-A1 recall ratings above 0, with 70% rating their MDP-A1 at 3 or more. In contrast, only 22% provided D-VAS instant ratings of 3 or higher. The median MDP-A1 score was 4.0 [2.0–6.0]. “Air hunger” was the most frequently selected sensory descriptor.

**Conclusions:**

Persistent dyspnea remains frequent and intense among patients being discharged after an acute respiratory episode.

**Supplementary Information:**

The online version contains supplementary material available at 10.1186/s12890-025-03923-3.

## Background

Unplanned hospitalizations for respiratory-related issues are prompted by vital or functional threats that requires specialized care and close monitoring. The indications for these admissions are driven by severity criteria related to the acute episode, the underlying chronic condition, or both [1–3]. While criteria-based discharge is less established, clinical criteria for discharge (CCDs) have been proposed in specific cases. These may be paired with discharge care bundles aimed at reducing length of stay, readmissions, or mortality, particularly following an exacerbation of chronic obstructive pulmonary disease (COPD) [4–6]. CCDs define the “*minimum physiological*,* therapeutic*,* and functional status the patient needs to achieve before discharge*.” [7].

Readmissions, length of stay, and mortality are easy to measure and have clear economic relevance. However, this relevance is less apparent to patients, who are primarily concerned with the symptoms they experience, dyspnea being a particularly distressing source of suffering. Following an acute respiratory episode, remaining dyspneic when one’s health is deemed sufficiently improved for discharge—which defines persistent dyspnea [8]—is likely to be anxiety-inducing. This may contribute to post-traumatic stress manifestations, which are not uncommon after severe COPD exacerbations [9, 10].

Despite its potential clinical relevance, dyspnea at the time of hospital discharge has not been specifically described. This exploratory study tests the primary hypothesis that a significant proportion of patients admitted to a pulmonology ward for an acute respiratory episode remain severely dyspneic at discharge. It also tests the secondary hypothesis that the prevalence and severity of “discharge dyspnea” are underestimated by a standard unidimensional, instant-based assessment tool, such as a dyspnea visual analogue scale, compared to a recall-based multidimensional assessment tool, the Multidimensional Dyspnea Profile (MDP) [11].

## Methods

### Ethical approval

This research received external approval from the *Comité de Protection des Personnes Île-de-France* III, Paris area, France, and all patients provided written informed consent to participate.

### Setting and study design

This observational study was conducted prospectively over 10 weeks at a single center (a 25-bed respiratory medicine ward within the 1,600-bed Pitié-Salpêtrière tertiary university hospital in Paris, France). Inclusion criteria were: (1) hospitalization for at least 24 h for any respiratory indication; (2) a dyspnea rating upon admission of 3 or higher on the MDP-A1 score (breathing discomfort evaluated on a 0–10 ordinal scale from “no discomfort” to “unbearable,” see below). Non-inclusion criteria were: (1) being a minor or under legal guardianship; (2) obvious cognitive improvement as assessed clinically during initial patient contact, based on observable disorientation, confusion, or inability to follow simple instructions (no formal cognitive screening, in line with the pragmatic, observational design). (3) aphasia or severe dysphasia; and (4) insufficient command of French. Of note, discharge criteria at our institution follow national guidelines, which primarily address chronic respiratory disease exacerbations but are applied more broadly for lack of specific alternatives [12]. These include clinical stability (normalized vitals, SpO₂ ≥ 88% in ambient air, stable blood gases), appropriate pharmacological management (oxygen when needed), patient education, and operational discharge follow-up strategy.

### Evaluation of dyspnea

#### Routine nurse evaluation

Dyspnea was assessed in an instant, unidimensional manner using a 10-cm visual analog scale (D-VAS, from 0: no respiratory discomfort to 10: maximal respiratory discomfort imaginable) by the nurses responsible for patient care. This evaluation followed the department’s routine protocol, with three systematic D-VAS measurements taken per day, coinciding with vital signs readings.

#### Research evaluation

Dyspnea was also assessed using the Multidimensional Dyspnea Profile (MDP) [11] within 24 h after hospital admission and within the 24 h before discharge. The MDP includes 11 items rated on 0–10 ordinal scales. One item quantifies respiratory discomfort, or dyspnea unpleasantness (MDP-A1). Five items identify and quantify sensory qualities of dyspnea (excessive breathing effort, air hunger, chest constriction, need to concentrate on breathing, and breathing a lot), which can be combined into a sensory quality score (MDP-SQ, maximum 50). Another five items describe the emotional qualities of dyspnea (anxiety, depression, frustration, fear, anger), forming an emotional response score (MDP-A2, maximum 50). MDP items can be grouped by dimensions (sensory –MDP-SQ– and affective –MDP-A1 + MDP-A2–) or by domains (immediate perception domain –MDP-SQ + MDP-A1– and emotional domain –MDP-A2–).

Participants are asked to focus on a specific event or period. In this study, this referred to the worst dyspneic episode experienced on the study day, whether at rest or with minimal effort (e.g., transferring from bed to chair, dressing, or going to the sink). If the worst episode on the admission day occurred during an effort, patients were asked to evaluate the same effort on the discharge day.

### Follow-up

Patients’ vital and health status at 90 days post-discharge was assessed using the French publicly accessible national vital statistics database (Institut National de la Statistique et des Études Économiques, INSEE, France) and available medical records.

### Stratification of the study population according to MDP-A1 evolution

The participating patients were stratified into three subgroups (“improved,” “unchanged,” or “worsened”) based on the admission-to-discharge difference in their MDP-A1 scores, with an improvement threshold of −1 point and a worsening threshold of + 1 point on the D-VAS scale.

### Statistical analysis

This study was conducted with a convenience sample, without a formal sample size estimation. Statistical analyses were performed using Prism^®^ v10 (Graphpad, CA, USA). Dichotomous variables were expressed as numbers and percentages and compared using McNemar’s test with continuity correction for paired data and Fisher’s exact test for unpaired data. Continuous data, generally not normally distributed, were summarized as median and interquartile range (median [IQR]). Paired comparisons between admission and discharge were conducted using Wilcoxon’s matched pairs signed rank test, while unpaired comparisons were performed using the Mann-Whitney test. Comparisons among the three MDP-A1 evolution subgroups were made using the Kruskal-Wallis test. Readmission and survival data were analyzed with the Kaplan-Meier method and compared using the log-rank test. The significance level was set at *p* = 0.05, with Holm-Sidak correction for multiple comparisons when appropriate.

## Results

### Study population

**Fig. 1:**
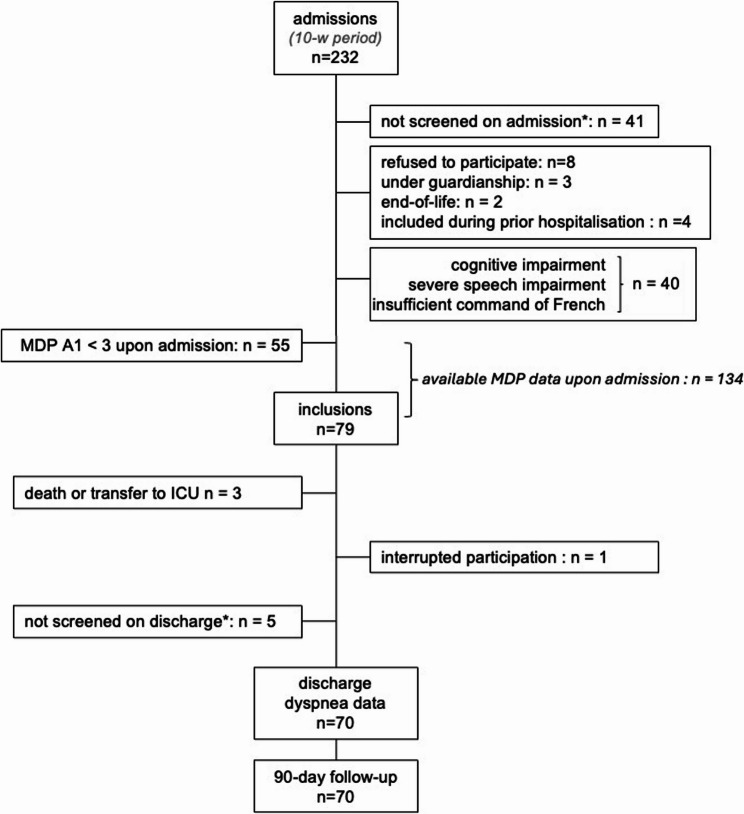
Study flow chart. MDP, Multidimensional Dyspnea Profile; MDP-A1: dyspnea unpleasantness rating (0–10 numerical rating scale); ICU: intensive care unit

Among the 232 patients admitted to the ward during the study period, 134 provided MDP data upon admission, of whom 79 (58.9%) had an MDP-A1 rating of ≥ 3 (Fig. 1). Seventy patients (age 63 [53–71]; 65% men, 35% women; 33% on long-term oxygen therapy or noninvasive home mechanical ventilation) were included in the final analysis (see reasons for attrition in Fig. 1). All patients (described in Table 1) received etiopathogenic treatments tailored to their physiological status and underlying diagnosis, in accordance with clinical guidelines and department protocols. None were prescribed symptomatic dyspnea-relieving treatments during their stay.

**Table 1:** Description of the study population and of the dyspnea evolution subgroups. Continuous variables are summarized as median[interquartile range]. Dichotomous values are provided as number and percentage

		Overall study population *n* = 70	MDP-A1-improved subgroup *n* = 46	MDP-A1-unchanged subgroup *n* = 16	MDP-A1-worsened subgroup *n* = 8
Age (years)		63 [53–71]	62 [52–71]	65 [60–72]	65 [58–72]
Sex (*n*/%)					
Men		46 (65%)	31 (67%)	10 (22%)	5 (11%)
Women		24 (35%)	15 (62.5%)	6 (25%)	3 (12.5%)
Body mass index (kg.m^−2^)		24 [21–29]	24 [22–28]	24 [21–31]	25 [20–31]
Reason for hospital admission°					
COPD exacerbation		21 (30%)	12 (57%)	5 (24%)	4 (19%)
Asthma exacerbation		5 (7%)	5 (100%)	0	0
Pleuritis/pneumothorax		12 (17%)	8 (67%)	3 (25%)	1 (8%)
Respiratory complication of neuromuscular disorders		6 (8.5%)	4 (67%)	2 (33%)	0
Pneumonia		7 (10%)	6 (86%)	0	1 (14%)
Hemoptysis		5 (7%)	3 (60%)	1 (20%)	1 (20%)
Pulmonary embolism		3 (4%)	0	3 (100%)	0
Thoracic mass or nodule		3 (4) %	2 (67%)	1 (33%)	0
Other		8 (11.5%)	6 (75%)	1 (12.5%)	1 (12.5%)
Known underlying diseases*					
Chronic heart disease		16 (23%)	12 (75%)	3 (19%)	1 (6%)
Lung cancer		18 (26%)	13 (72%)	5 (28%)	0
COPD		24 (34%)	14 (58%)	6 (25%)	4 (17%)
Interstitial lung disease		5 (7%)	4 (80%)	1 (20%)	0
Neuromuscular disease**		8 (11%)	6 (75%)	2 (25%)	0
Bronchiectasis		6 (8%)	3 (50%)	0	3 (50%)
Asthma		11 (15%)	9 (82%)	0	2 (18%)
Treatments recorded on admission					
Long term oxygen therapy		9 (13%)	5 (56%)	2 (22%)	2 (22%)
Home noninvasive mechanical ventilation		14 (20%)	10 (71%)	2 (14%)	2 (14%)
Benzodiazepins		12 (17%)	7 (58%)	4 (33%)	1 (8%)
Opiates***		4 (6%)	2 (50%)	1 (25%)	1 (25%)
Hospital length of stay (days) °°		7 [5–11]	9 [7–11]	5.5 [3-7.3]	8.5 [6-17.5]

*possibly more than one per patient, as follows : 8 ot the 24 COPD patients, 3 of the 18 cancer patients, 1 amyotrophic lateral sclerois patient and one interstitial lung disease patient had a know associated cardiac disease (ischemic in most cases)

**mostly amyotrophic lateral sclerosis

***prescribed for pain management in all cases

°Twenty-four patients were admitted directly from home, 19 from the hospital emergency department, 21 from a critical care unit, 5 from other departments, and 1 from a rehabilitation facility

°°None of the patients died during their stay and one was transferred to a critical care facility. Forty-seven patients were discharged to home, 14 to a pulmonary rehabilitation facility (COPD in all cases), and 9 to another medicine department

### Dyspnea upon admission

MDP-A1 on admission was 6.5 [5.0–8.0]. As per the inclusion criteria, all seventy patients (100%) had MDP-A1 ratings above 0, with 5 patients (7%) rating MDP-A1 at “3” and 65 patients (93%) rating it at “4 or above.” The concurrent D-VAS was 4.0 [0.0–6.0] (*p* < 0.001).

**Fig. 2:**
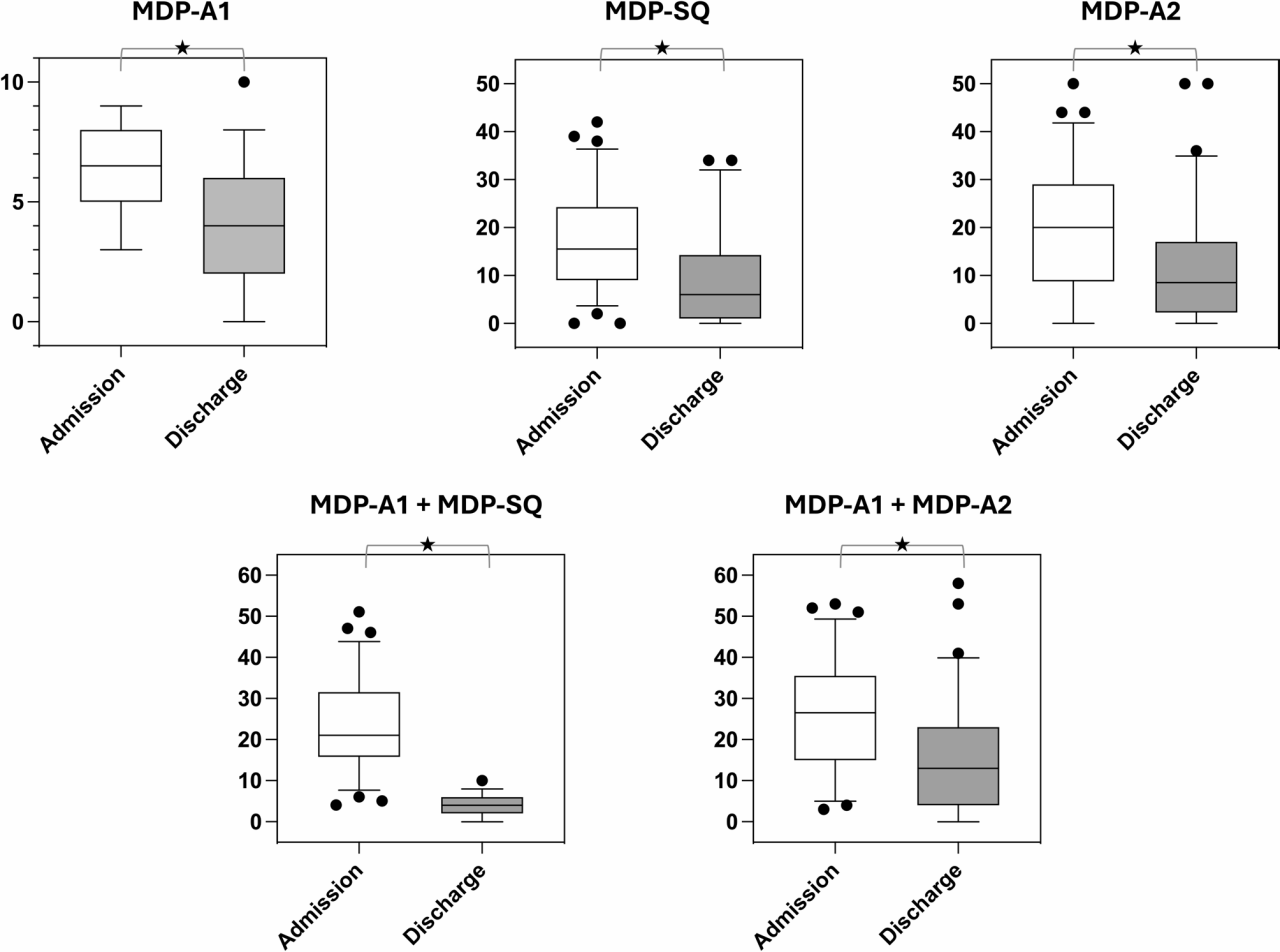
Comparision of the Multidimensional Dyspnea Profile aggregated scores between admission and discharge. MDP-A1, intensity of dyspnea unpleasantness; MDP-SQ, sum of sensory qualifier ratings, also sensory dimension of dyspnea; MDP-A2, sum of emotional ratings, also dyspnea emotional domain; MDP-A1 + SQ, immediate dyspnea perception domain; MDP-A1 + MDP-A2, affective dimension of dyspneaMDP-

The MDP-SQ was 15.5 [9.3–24.0], and the MDP-A2 was 20.0 [9.3–28.8] (Fig. 2; Table 2). Among MDP-SQ descriptors, “air hunger” was most frequently selected, applying best for 30 patients (43%) and receiving the highest ratings (6.0 [3.0–8.0]) (Table 2).

**Table 2:** Detailed multidimensional dyspnea profile values upon admission and discharge. Values in median [interquartile range]

	Admission	Discharge	*p*	Below significance z	Holm-Sidak’s adjusted *p*
MDP-A1	6.5 [5.0–8.0]	4.0 [2.0–6.0]	**< 0.0001**	**Yes**	**0.0025**
MDP-SQ	16.0 [9.0–24.0]	6.0 [1.0–14.0]	**< 0.0001**	**Yes**	**0.0025**
MDP-SQ1 (excessive breathing effort)	2.5 [0.0-6.3]	0.0 [0.0–3.0]	**0.0020**	**Yes**	**0.0218**
SQ1 applies	36 (51.43%)	27 (38.57%)	0.1096	No	0.5364
SQ1 best applies	7 (10.00%)	11 (15.71%)	0.0455	No	0.3110
MDP-SQ2 (air hunger)	6.0 [3.0–8.0]	2.0 [0.0–6.0]	**< 0.0001**	**Yes**	**0.0025**
SQ2 applies	58 (82.86%)	36 (51.43%)	**0.0001**	**Yes**	**0.0025**
SQ2 best applies	30 (42.86%)	19 (27.14%)	0.1456	No	0.5364
MDP-SQ3 (chest constriction)	4.0 [0.0–6.0]	0.0 [0.0-3.3]	**< 0.0001**	**Yes**	**0.0025**
SQ3 applies	43 (61.43%)	27 (38.57%)	**0.0014**	**Yes**	**0.0194**
SQ3 best applies	15 (21.43%)	12 (17.14%)	0.7237	No	0.9237
MDP-SQ4 (need to concentrate on breathing)	0.0 [0.0–6.0]	0.0 [0.0–3.0]	0.1040	No	0.5364
SQ4 applies	31 (44.29%)	26 (37.14%)	0.3588	No	0.7364
SQ4 best applies	7 (10.00%)	4 (5.71%)	0.7237	No	0.9237
MDP-SQ5 (breathing a lot)	0.5 [0.0–6.0]	0.0 [0.0–2.0]	**0.0005**	**Yes**	**0.0075**
SQ5 applies	34 (48.57%)	21 (30.00%)	0.0164	No	0.1383
SQ5 best applies	6 (8.57%)	7 (10.00%)	0.1336	No	0.5364
MDP-A2	20.0 [8.8–29.0]	8.5 [2.3–17.0]	**< 0.0001**	**Yes**	**0.0025**
MDP-A2-1 (anxiety)	3.5 [0.0–8.0]	0.0 [0.0–5.0]	0.0144	No	0.1350
MDP-A2-2 (depression)	5.0 [2.0–8.0]	2.0 [0.0–5.0]	**< 0.0001**	**Yes**	**0.0025**
MDP-A2-3 (frustration)	5.0 [0.0–7.0]	0.0 [1.0–5.0]	**0.0014**	**Yes**	**0.0194**
MDP-A2-4 (fear)	0.0 [0.0–6.0]	0.0 [0.0-3.3]	**0.0014**	**Yes**	**0.0194**
MDP-A2-5 (anger)	4.5 [0.0-7.3]	0.0 [0.0–4.0]	**< 0.0001**	**Yes**	**0.0025**
MDP-A1 + MDP-SQ	21.0 [16.0–32.0]	4.0 [2.0–6.0]	**< 0.0001**	**Yes**	**0.0025**
MDP-A1 + MDP-A2	27.0 [15.0–36.0]	13.0 [4.0–23.0]	**< 0.0001**	**Yes**	**0.0025**

*MDP-A1* intensity of respiratory discomfort or dyspnea unpleasantness, MDP-*SQ* sum of MDP-SQx ratings, also sensory dimension, *SQx* sensory qualifiers, *A2-x* emotional descriptors, *MDP-A2* sum of A2-x ratings, also emotional domain, *MDP-A1 + MDP-A2* affective dimension, *MDP-A1 + MDP-SQ* immediate perception domain

### Dyspnea upon discharge

**Fig. 3:**
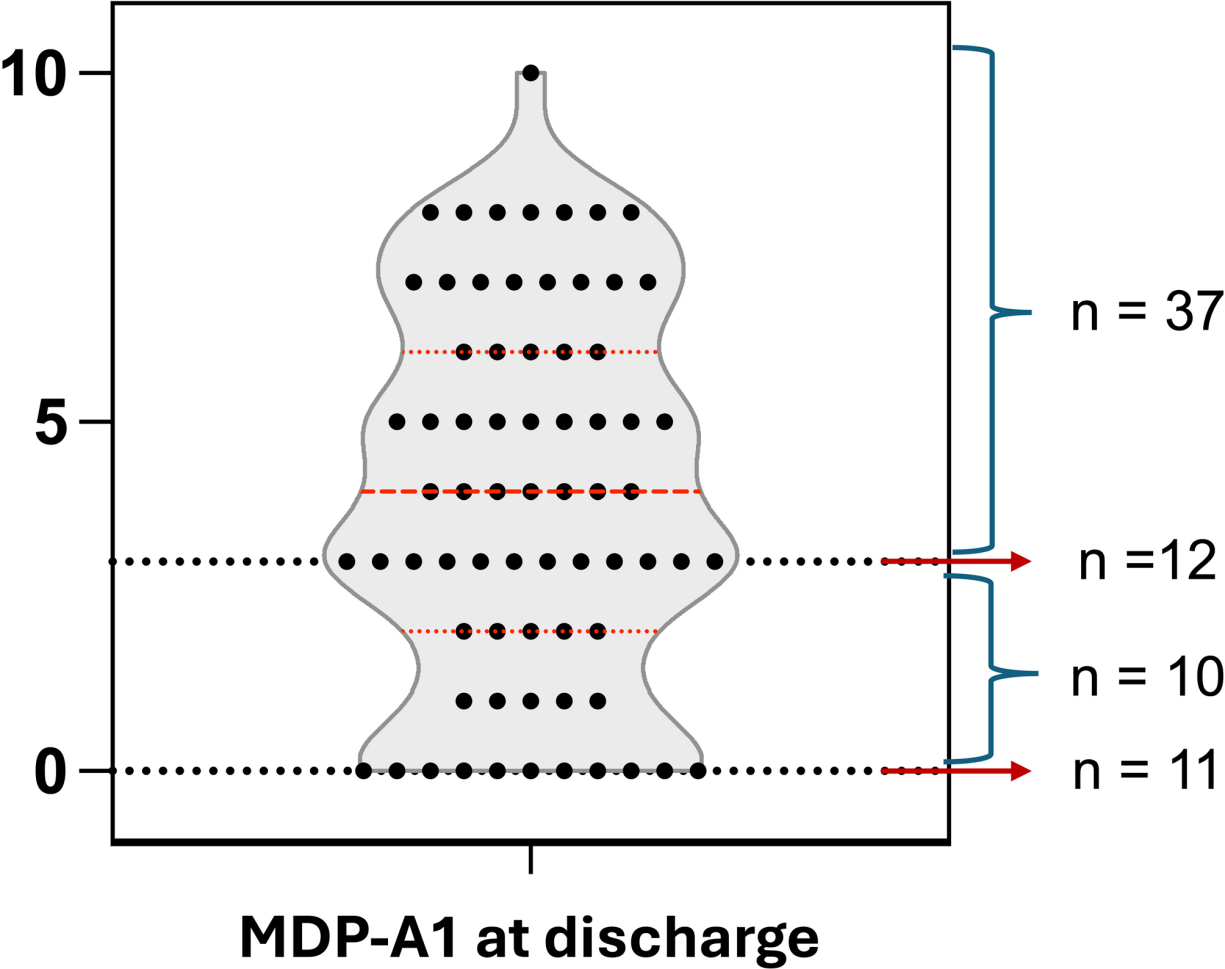
Distribution of dyspnea unpleasantness ratings (MDP-A1, 0–10 numerical rating scale) upon hospitalisation discharge

MDP-A1 on discharge was 4.0 [2.0–6.0]. Fifty-nine patients (84%) had MDP-A1 ratings above 0. Twenty-one patients (30%) provided MDP-A1 ratings below 3 (asthma exacerbation, *n* = 5; COPD exacerbation, *n* = 4; heart failure, *n* = 4; neuromuscular diseases, *n* = 3; lung cancer, *n* = 3; interstitial lung disease, *n* = 2). The remaining 70% had MDP-A1 ratings of 3 or higher (Fig. 3). The concurrent D-VAS was 0.0 [0.0–2.0] (*p* < 0.001), with 22% of patients providing D-VAS ratings of 3 or higher.

The MDP-SQ was 6.0 [1.0–14.0], and the MDP-A2 was 8.5 [2.3–17.0] (Fig. 2; Table 2). “Air hunger” remained the most frequently selected MDP-SQ descriptor, best applying to 19 patients (21%) and receiving the highest ratings of 2.0 [0.0–6.0] (Table 2).

### Evolution of dyspnea during the hospital stay

At the population level, all dyspnea indicators were significantly lower at the end of the hospital stay than at its beginning (Table 2; Fig. 2). This reduction was primarily driven by a subgroup of 46 patients whose respiratory discomfort improved, as indicated by discharge MDP-A1 ratings that were at least one point lower than their admission MDP-A1 ratings. Sex, age, and body mass index did not influence MDP-A1 dynamics during the hospital stay. The presence of bronchiectasis was significantly associated with stable or worsening MDP-A1 scores (*p* = 0.0058), while pulmonary embolism as an admission diagnosis was significantly associated with MDP-A1 stability (*p* = 0.0050). Among COPD patients, “air hunger” (SQ2) was the predominant symptom upon admission, but “excessive breathing effort” (SQ1) became predominant at discharge. In neuromuscular patients, “air hunger” (SQ2) remained predominant at both admission and discharge. For asthma patients, “chest constriction” (SQ3) was the predominant symptom at admission and remained so among those who continued to report dyspnea at discharge. Notably, MDP-A2 (the affective dimension of dyspnea) was significantly higher in patients whose condition did not improve or worsened during the hospital stay, with depression, anxiety, and frustration being the predominant emotions (Table S1 in the electronic supplement).

### Relationships between dyspnea and clinical outcomes

At the population level, the median hospital stay was 7 [5–11] days. Patients in the MDP-A1-unchanged subgroup had a statistically significantly shorter hospital stay than other patients (5.5 [3.0–7.3] days, *p* = 0.0333), despite having admission MDP-A1 ratings comparable to those of the other subgroups (Table S1). Changes in MDP-SQ and MDP-A2 were not associated with hospitalization duration.

**Fig. 4:**
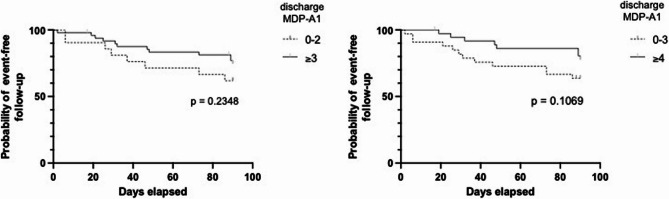
Kaplan-Meier event-free survival curves according to MDP-A1 discharge ratings (left: 0–2 vs. ≥3; right: 0–3 vs. ≥ 4)

Discharge settings are provided in Table 1. After discharge, sixteen patients (23%) were readmitted in an unplanned manner within 90 days of discharge, with a median interval of 30.5 [17.25–47.75] days. Their MDP-A1, MDP-SQ, and MDP-A2 scores (at both admission and discharge) did not differ significantly from those of patients who were not readmitted. There was no statistically significant excess of any specific admission motives, although 6 of the 16 readmitted patients had cancer. Seven patients died during the follow-up period, with 4 deaths occurring independently of readmission (cumulative readmissions and deaths, *n* = 20). A discharge MDP-A1 of 3 or more, or of 4 or more, was not associated with a higher risk of readmission or death (Fig. 4). The evolution of dyspnea between admission and discharge was also not associated with an increased risk of readmission or death.

## Discussion

This study shows that patients admitted to a respiratory medicine ward for an acute respiratory episode unsurprisingly report very high levels of dyspnea; that dyspnea generally improves during the hospital stay but does so inconsistently; and that a majority of patients remain clinically significantly dyspneic upon discharge. The study also reveals a substantial discrepancy between an immediate (D-VAS) and a recall-based (MDP-A1) assessment in capturing patients’ experiences. Of major clinical relevance, this suggests that relying on immediate D-VAS to tailor post-discharge management in respiratory patients carries the risk of undertreatment.

This study appears to be the first to focus on the dyspnea of patients hospitalized for an acute respiratory episode in a specialized facility and, uniquely, on dyspnea at the time of discharge. This novelty limits the possibility of direct comparisons. Meek et al. [13] studied 151 patients admitted to the emergency room for various respiratory conditions and reported MDP-A1 ratings slightly lower than those in our patients (5.1 ± 2.6), while other MDP indicators were roughly comparable. Dyspnea was almost never fully resolved by the time of emergency room discharge. Stevens et al. [14] conducted a multidimensional assessment of dyspnea in a general inpatient population of 156 adults who rated their dyspnea as 4 or more on a unidimensional 0–10 scale (from “no dyspnea” to “unbearable dyspnea”) on the day of admission. These patients provided MDP-A1 ratings of 4.3 ± 2.2 at the moment of their first research interview versus 7.8 ± 2.3 when asked about their worst episode during the previous 24 h, a figure closely resembling the ratings of our patients on admission. In both studies, air hunger was the predominant sensation. Most patients in the study by Stevens et al. [14] reported a decrease in dyspnea intensity over time, buth their evaluation spanned only a short portion of the hospital stay and did not address the time of discharge. Of note, the persistence of dyspnea after an acute respiratory episode is well-documented and not unexpected. For instance, exercise-limiting dyspnea frequently remains a factor after acute pulmonary embolism [15]. Dyspnea also persists after acute cardiac episodes, significantly impacting quality of life [16]. This phenomenon is particularly prevalent and intense in patients following a COPD exacerbation [17, 18]. We did not observe significant associations between discharge MDP-A1 ratings and readmission or death within the following 90 days. This finding does not align with existing literature on the prognostic value of dyspnea [19–21]. This discrepancy may be attributed to the heterogeneity of our limited sample size, which may have limited the ability of dyspnea to function as an effective prognostic indicator.

A prominent feature of our study is the discrepancy between the results of instant dyspnea evaluation (D-VAS data) and recalled dyspnea evaluation (MDP-A1 data) on the day of discharge. Beyond a clear difference in ratings (D-VAS: 0.0 [0.0–2.0] vs. MDP-A1: 4.0 [2.0–6.0]), this discrepancy was striking in terms of the number of patients affected: 70% of patients provided MDP-A1 ratings of 3 or more, compared to only 22% for D-VAS. Stevens et al. [14]observed a similar discrepancy in admission data when comparing MDP-A1 ratings for “dyspnea at the time of the research interview” and for “worst dyspnea episodes during the preceding 24 hours.” Such differences are unsurprising, as instant dyspnea evaluations are generally performed while patients are at rest (e.g., during vital sign readings by a nurse or a research interview), which is when dyspnea is most likely to improve with treatment. In our study, it was common to hear patients state that they felt much improved and not breathless at such moments, only to exhibit anticipatory distress when asked about performing tasks like going to the toilet or attending physiotherapy sessions.

This study has limitations. It involves a small population and was conducted at a single center, so it should be viewed as a preliminary snapshot identifying a potential problem rather than providing definitive conclusions. Of note however, the population size falls in the middle range of populations used to multidimensionally characterize dyspnea in other settings [13, 14, 22, 23]. Our study population is also highly heterogeneous, which precludes any disease-specific conclusions. However, this heterogeneity, resulting from the study’s pragmatic design, underscores that the identified issue (persistent dyspnea) may be general in nature. Our choice of an MDP-A1 rating of 3 or more as the main inclusion criterion could be subject to criticism. We selected a threshold of “3” instead of “4”—a value often used to indicate “clinically relevant” dyspnea—because the figure of “4” lacks objectively demonstrated justification. For instance, 30% of patients reporting an MDP-A1 rating of 3 consider their dyspnea unbearable [14]. Moreover, patients with MDP-A1 ratings from 1 to 3 on admission have a significantly higher mortality risk compared to those who report no dyspnea [24]. Finally, for those of our patients with underlying chronic respiratory diseases, we cannot provide a comparison between the present discharge dyspnea data and baseline dyspnea descriptions. The MDP ratings observed at discharge are generally lower than baseline MDP ratings previously reported in stable COPD or amyotrophic lateral sclerosis patients [22, 23]. However, this finding is difficult to interpret due to the lack of comparability in the MDP recall period across studies. Besides these limitations, it is important to point out the innovative nature of multidimensionally portraying dyspnea with the MDP. This is true even though in this study the MDP was applied by the research team and not the routine nursing team, which would require specific training in addition to the DVAS training.

## Conclusions

Despite study limitations, we believe that our observations call for caution regarding persistent dyspnea in patients discharged at the end of an acute respiratory episode. Given the corresponding burden, any patient in this situation may warrant symptomatic interventions, irrespective of the underlying diagnosis. Our findings support the need for further studies to assess the impact of discharge dyspnea on patient-centered outcomes in the weeks or months following a respiratory-related hospitalization, with particular emphasis on post-traumatic manifestations. In this context, introducing symptomatic therapeutic measures alongside etiopathogenic treatments at an early stage should be considered and warrants specific investigation. Meanwhile, it is important to recognize that evaluating dyspnea in a recall manner should reduce the risk of overlooking the respiratory burden experienced by patients upon discharge after an acute respiratory episode, be it with a specifically recall tool like the MDP or with a simple DVAS, depending on each team’s habits and training.

## Supplementary Information

Supplementary Material 1.

## Data Availability

Individual participant data that underlie the results reported in this article will be shared after deidentification upon reasonable request to the corresponding author (thomas.similowski@sorbonne-universite.fr).

## Abbreviations

MDP-A2: Emotional response score of the multidimensional dyspnea profile
CCD: Criteria for discharge
COPD: Chronic obstructive pulmonary disease
D-VAS: Dyspnea visual analog scale
MDP-A1: Breathing discomfort/dyspnea unpleasantness on a 0-10 numerical rating scale
MDP: Multidimensional dyspnea profile
MDP-SQ: Sensory quality score of the multidimensional dyspnea profile
