# Supplementary material for: Is ‘better’ enough? Prevalence and multidimensional portrait of persistent dyspnea upon discharge from a respiratory medicine ward: a prospective, single-center observational study
Source: BMC Pulm Med. 2025 Oct 6;25:452. doi: 10.1186/s12890-025-03923-3 (PMC12502202; doi:10.1186/s12890-025-03923-3)
Supplement: Supplementary file 1 — Supplementary Material 1. [file 12890_2025_3923_MOESM1_ESM.docx]

***Electronic supplement to***

*Is better enough? Persistent dyspnea on respiratory medicine discharge: prospective observations*

Capucine MORÉLOT-PANZINI, MD, PhD (1,2)*, Safaa NEMLAGHI, MD (2)* (* co-first authors), Morgane FAURE (2), Laure SERRESSE (1,3), Thomas SIMILOWSKI, MD, PhD (1, 4).

*1. Sorbonne Université, INSERM, UMRS1158 Neurophysiologie Respiratoire Expérimentale et Clinique, F-75005 Paris, France*

*2. AP-HP, Groupe Hospitalier Universitaire APHP-Sorbonne Université, hôpital Pitié-Salpêtrière, Service de Pneumologie (Département R3S), F-75013 Paris, France*

*3. AP-HP, Groupe Hospitalier Universitaire APHP-Sorbonne Université, Service de Soins*

*Palliatifs, Soins d'Accompagnement et Soins de Support" F-75013 Paris, France.*

*4. AP-HP, Groupe Hospitalier Universitaire APHP-Sorbonne Université, hôpital Pitié-Salpêtrière, Département R3S, F-75013 Paris, France*

**Table S1.** Dyspnea evaluation upon admission and discharge according to the pattern of evolution of Multidimensional Dyspnea Profile A1 ratings during the hospital stay.

|  | | Overall study population  n=70 | A1-improved subgroup  n=46 | A1-unchanged subgroup  n=16 | A1-worsened subgroup  n=8 | p* |
| --- | --- | --- | --- | --- | --- | --- |
| **Admission** | | | | | | |
| D-VAS | | 4.0 [0.0-6.0] | 5.0 [0.0-6.0] | 0.0 [0.0-2.8] | 6.0 [5.0-7.0] | 0.0241 |
| **MDP-A1** | | 6.5 [5.0-8.0] | 7.0 [5.0-8.0] | 7.0 [5.0-8.0] | 4.5 [3.8-5.3] | 0.0262 |
| **MDP-SQ** | | 15.5 [9.3-2.4] | 17 [9.3-24] | 13.5 [9.5-26] | 15.0 [9.8-20.0] | ns |
|  | MDP-SQ1 (excessive breathing effort) | 2.5 [0.0-6.0] | 3.5 [0.0-6.0] | 0.5 [0.0-7.0] | 2.5 [0-5.25] | ns |
|  | MDP-SQ2 (air hunger) | 6.0 [3.0-8.0] | 6.0 [2.0-8.0] | 7.0 [3.75-8.0] | 5 [3-8] | ns |
|  | MDP-SQ3 (chest constriction) | 4.0 [0.0-6.0] | 4.0 [0.0-6.0] | 4.0 [0.0-6.25] | 4 [0-6.25] | ns |
|  | MDP-SQ4 (need to concentrate on breathing) | 0.0 [0-5.75] | 0.0 [0.0-6.0] | 1.5 [0.0-6.25] | 0.0 [0.0-4.25] | ns |
|  | MDP-SQ5 (breathing a lot) | 0.5 [0-5.75] | 2.0 [0.0-6.75] | 0.5 [0.0-5.25] | 0.0 [0.0-3.5] | ns |
| **MDP-A2** | | 20 [9.3-28.8] | 21.0 [11.0-29.0] | 20.5 [10.3-30] | 7.5 [4.5-17.3] | ns |
|  | MDP-A2-1 (anxiety) | 5.0 [2.0-8.0] | 5.5 [3.0-8.0] | 4.5 [0.0-7.25] | 2.0 [1.5-5.0] | ns |
|  | MDP-A2-2 (depression) | 3.5 [0.0-7.75] | 4.5 [0.0-8.0] | 2.5 [0.0-5.25] | 2.5 [0.0-3.25] | ns |
|  | MDP-A2-3 (frustration) | 5.0 [0.0-7.0] | 5.0 [0.0-7.0] | 5.0 [0.0-8.0] | 1.0 [0.0-3.75] | ns |
|  | MDP-A2-4 (fear) | 4.5 [0.0-7.0] | 5.0 [0.0-7.0] | 4.0 [0.0-8.0] | 1.0 [0.0-4.0] | ns |
|  | MDP-A2-5 (anger) | 0.0 [0.0-5.75] | 0.0 [0.0-5.0] | 4.0 [0.0-7.25] | 0.0 [0.0-1.5] | ns |
| **Discharge** | | | | | | |
| D-VAS | | 0.0 [0.0-2] | 0.0 [0.0-0.0] | 0.0 [0.0-4.8] | 3.0 [0.0-5.0] | 0.0209 |
| **MDP-A1** | | 4.0 [2.0-6.0] | 3.0 [1.0-4.0] | 7.0 [5.0-8.0] | 6.5 [5.8-8.0] | <0.0001 |
| **MDP-SQ** | | 6.0 [1.3-14.0] | 4.0 [0.0-7.0] | 15.5 [9.3-26] | 14.0 [9.0-21.0] | <0.0001 |
|  | MDP-SQ1 (excessive breathing effort) | 0.0 [0.0-3.0] | 0.0 [0.0-2.0] | 2.0 [0.0-4.5] | 0.0 [0.0-5.5] | ns |
|  | MDP-SQ2 (air hunger) | 2.0 [0.0-6.0] | 0.0 [0.0-2.0] | 6.0 [3-7.25] | 6.0 [4.75-8.0] | <0.0001 |
|  | MDP-SQ3 (chest constriction) | 0.0 [0.0-3.0] | 0.0 [0.0-2.0] | 4.0 [0.0-6.25] | 0.0 [0.0-6.0] | 0.0351 |
|  | MDP-SQ4 (need to concentrate on breathing) | 0.0 [0.0-3.0] | 0.0 [0.0-1.0] | 1.5 [0.0-6.25] | 1.5 [0.0-5.75] | 0.0391 |
|  | MDP-SQ5 (breathing a lot) | 0.0 [0.0-2.0] | 0.0 [0.0-0.0] | 3.0 [0.75-5.25] | 0.0 [0.0-1.25] | <0.0001 |
| **MDP-A2** | | 8.5 [3.0-17] | 4 [0.0-13.5] | 15.5 [6.3-24.0] | 15.5 [13.0-20.0] | 0.0055 |
|  | MDP-A2-1 (anxiety) | 2.0 [0.0-5.0] | 0.0 [0.0-3.75] | 5.0 [1.5-7.0] | 2.5 [0.0-5.0] | 0.0371 |
|  | MDP-A2-2 (depression) | 0.0 [0.0-5.0] | 0.0 [0.0-3.0] | 2.0 [0.0-6.25] | 5.5 [4.75-6.5] | 0.0007 |
|  | MDP-A2-3 (frustration) | 1.0 [0.0-5.0] | 0.0 [0.0-4.0] | 2.0 [0.0-8.0] | 4.0 [0-5.25] | ns |
|  | MDP-A2-4 (fear) | 0.0 [0.0-4.0] | 0.0 [0.0-2.0] | 0.5 [0.0-7.25] | 3.5 [0.0-5.25] | ns |
|  | MDP-A2-5 (anger) | 0.0 [0.0-3.0] | 0.0 [0.0-2.0] | 0.0 [0.0-5.0] | 1.0 [0.0-2.5] | ns |

A1. intensity of respiratory discomfort or dyspnea unpleasantness. SQ. sum of SQx ratings. also sensory dimension.SQx. sensory qualifiers. A2-x. emotional descriptors. A2. sum of A2-x ratings. also emotional domain.

* Kruskall-Wallis non-parametric analysis of variance
